# Mental Health and Functional Outcomes in Young Adulthood of Children With Psychotic Symptoms: A Longitudinal Cohort Study

**DOI:** 10.1093/schbul/sbz069

**Published:** 2019-07-30

**Authors:** Antonella Trotta, Louise Arseneault, Avshalom Caspi, Terrie E Moffitt, Andrea Danese, Carmine Pariante, Helen L Fisher

**Affiliations:** 1 Social, Genetic & Developmental Psychiatry Centre, Institute of Psychiatry, Psychology & Neuroscience, King’s College London, London UK; 2 Tony Hillis Unit, Lambeth Hospital, South London and Maudsley NHS Foundation Trust, London, UK; 3 Department of Psychology and Neuroscience, Duke University, Durham, NC; 4 Department of Psychiatry and Behavioral Sciences, Duke University Medical School, Durham, NC; 5 Department of Child & Adolescent Psychiatry, Institute of Psychiatry, Psychology & Neuroscience, King’s College London, London UK; 6 National & Specialist CAMHS Clinic for Trauma, Anxiety and Depression, South London & Maudsley NHS Foundation Trust, London, UK; 7 Department of Psychological Medicine, Institute of Psychiatry, Psychology & Neuroscience, King’s College London, London UK

**Keywords:** adolescence, depression, follow-up, functioning, physical health, psychosis

## Abstract

**Background:**

Childhood psychotic symptoms have been associated with various psychiatric disorders in adulthood but their role as early markers of poor outcomes during the crucial transition to adulthood is largely unknown. Therefore, we investigated associations between age-12 psychotic symptoms and a range of mental health problems and functional outcomes at age 18.

**Methods:**

Data were used from the Environmental Risk Longitudinal Twin Study, a nationally representative birth cohort of 2232 twins born in 1994–1995 in England and Wales, followed to age 18 with 93% retention. Childhood psychotic symptoms were assessed in structured interviews at age 12. At age 18, study members’ mental health problems, functional outcomes, risky behaviors, and offending were measured using self-reports and official records.

**Results:**

Children with psychotic symptoms (*N* = 125, 5.9%) were more likely to experience a range of mental health problems in young adulthood than children without such symptoms. They were also more likely to be obese, smoke cigarettes, be lonely, be parents, and report a lower quality of life, but not more likely to commit crimes. Childhood psychotic symptoms predicted these poor outcomes over and above other emotional and behavioral problems during childhood. Nevertheless, twin analyses indicated that these associations were largely accounted for by shared family factors.

**Conclusions:**

Psychotic symptoms in childhood signal risk for pervasive mental health and functional difficulties in young adulthood and thus may provide a useful screen for an array of later problems. However, early psychotic symptoms and poor outcomes may be manifestations of shared environmental and genetic risks.

## Introduction

It has been almost two decades since we published one of the first studies providing direct evidence of a continuity of psychotic symptoms from childhood to adulthood.^1^ The initial report drew the attention of researchers to the significance of early psychotic symptoms and argued that such symptoms should be routinely assessed in child mental health clinical practice.^1^ Subsequent research^2–5^ has supported the finding that psychotic symptoms in childhood are a marker of developmental difficulties, including emotional, cognitive, and social deficits,^6^ as well as a risk factor for a range of other mental health and functional problems in adolescence and adulthood.^7–12^ However, despite the abundant research evidence, the recommendation to routinely assess children for symptoms of psychosis has not yet been implemented in clinical practice.

Here we seek to reignite interest in these early psychotic phenomena by highlighting the difficulties that affected children can experience as they enter adulthood. Problems during the transition to adulthood interfere with the attainment of important social and vocational goals leading to adverse socioeconomic consequences in later life.^13^ Therefore, we capitalized on data from a nationally representative birth cohort of twins to explore associations between childhood psychotic symptoms and a range of outcomes in areas critical to this stage of development. Specifically, we tested the hypothesis that psychotic symptoms at age 12 would predict a higher prevalence of mental health problems, worse social and occupational functioning, engaging in more risky behaviors, and higher rates of offending at age 18.

Guided by previous literature, we additionally tested whether childhood psychotic symptoms contributed to poor outcomes independently of individual and family characteristics, comorbid childhood psychopathology, and familial risk. First, we adjusted analyses for study member’s sex, their IQ at age 5, and their family’s socioeconomic status (SES), as previous studies have identified male gender,^14^ early cognitive impairment,^6,15^ and lower family SES^16^ as predictors of poorer outcomes in individuals with psychosis. Second, we attempted to disentangle the specific impact of childhood psychotic symptoms on poor outcomes in young adulthood, by adjusting for mental health problems that commonly co-occur with these early psychotic phenomena, namely depression, anxiety, conduct disorder, attention-deficit hyperactivity disorder (ADHD), and self-injurious behaviors,^2,6^ several of which have also been linked to poorer adult outcomes.^17–19^ Finally, we investigated whether childhood psychotic symptoms predicted poorer age-18 outcomes over and above shared familial risk factors. Having one or more biological parents with a history of psychosis has been associated with a greater risk of psychotic symptoms in offspring,^20^ and twin studies have suggested that both shared genetic and environmental familial risk factors contribute to variability in early psychotic phenomena.^21^ Moreover, familial risk factors implicated in the etiology of childhood psychotic symptoms, such as maltreatment and genetic susceptibility, have also been shown to predict poor functioning in young adulthood.^22,23^ To take into account familial risk, we initially adjusted associations for familial psychopathology. We then used a discordant twin approach by comparing young-adult outcomes between twins growing up in the same family, where one twin had experienced psychotic symptoms at age 12 and the other had not. Because these twins share most of their family-wide environment and (at least 50% of) their genes, these analyses control for the majority of the familial risk factors shared between members of a family.

## Methods

### Study Cohort

Participants were members of the Environmental Risk (E-Risk) Longitudinal Twin Study, which tracks the development of a birth cohort of 2232 British children. The sample was drawn from a larger birth register of twins born in England and Wales in 1994–1995.^24,25^ Briefly, the E-Risk sample was constructed in 1999–2000, when 1116 families (93% of those eligible) with same-sex 5-year-old twins participated in home visit assessments. This sample comprised 56% monozygotic (MZ) and 44% dizygotic (DZ) twin pairs; sex was evenly distributed within zygosity (49% male). E-Risk participants are representative of UK households across the spectrum of neighborhood socioeconomic conditions: 27.0% of E-Risk participants lived in “wealthy achiever” neighborhoods compared to 25.4% of households nationwide, 7.2% vs 11.5% lived in “urban prosperity” neighborhoods, 26.8% vs 27.4% lived in “comfortably off” neighborhoods, 13.2% vs 13.8% lived in “moderate means” neighborhoods, and 25.8% vs 21.2% lived in “hard-pressed” neighborhoods.^26^ E-Risk underrepresents urban prosperity neighborhoods because such households are likely to be childless.

Follow-up home visits were conducted when the children were aged 7 (98% participation), 10 (96%), 12 (96%), and 18 years (93%). Home visits at ages 5, 7, 10, and 12 years included assessments with participants as well as their mother (or primary caretaker); the home visit at age 18 included structured interviews only with the participants. Each twin participant was assessed by a different interviewer. The average age of the twins at the time of the age-18 assessment was 18.4 years (*SD* = 0.36); all structured interviews were conducted after the 18th birthday. There were no differences in SES assessed when the cohort was initially defined (χ ^2^ = 0.86, *P* = .65), age-5 IQ scores (*t* = 0.98, *P* = .33), or age-5 internalizing or externalizing behavior problems (*t* = 0.40, *P* = .69, and *t* = 0.41, *P* = .68, respectively), between those who did and did not take part at age 18.

The Joint South London and Maudsley and the Institute of Psychiatry Research Ethics Committee approved each phase of the study. Parents gave informed consent and twins gave assent between 5 and 12 years and then informed consent at age 18.

### Childhood Psychotic Symptoms

E-Risk families were visited by interviewers when children were aged 12. Each child was privately interviewed about 7 psychotic symptoms pertaining to delusions and hallucinations. Items and interviewer notes were assessed by a psychiatrist expert in schizophrenia, a psychologist expert in interviewing children, and a child and adolescent psychiatrist to verify the validity of the symptoms. This structured interview and coding procedure has been described in detail previously^6^ and in the supplementary materials. At age 12, 125 (5.9%) children were designated as experiencing at least 1 definite psychotic symptom. This is similar to the prevalence of psychotic symptoms in other community samples of children and adolescents.^2,27^ Note, only one of these children was diagnosed with a psychotic disorder.

### Young-Adult Psychopathology

During the age-18 interview, interviewers assessed participants’ mental health over the previous 12 months including depressive disorder (*n* = 414, 20.1%), generalized anxiety disorder (*n* = 153, 7.4%), alcohol dependence (*n* = 573, 27.8%), cannabis dependence (*n* = 124, 6.0%), dependence on other drugs (*n* = 20, 1.0%), and conduct disorder (*n* = 309, 15.0%) according to *Diagnostic and Statistical Manual of Mental Disorders, Fourth Edition* (*DSM-IV*) criteria,^28^ post-traumatic stress disorder (PTSD; *n* = 90, 4.4%) and ADHD (*n* = 171, 8.3%) according to *DSM-5* criteria.^29^ Other drugs included nonprescription use of stimulants, sedatives, cocaine/crack, painkillers, street opiates, club drugs, hallucinogens, and inhalants. Assessments were conducted in face-to-face structured interviews using the Diagnostic Interview Schedule.^30^

Each E-Risk participant was privately interviewed about 13 psychotic *experiences* occurring between ages 12 and 18. Seven items pertained to delusions and hallucinations (see earlier) and 6 items pertained to unusual experiences which drew on item pools since formalized in prodromal psychosis instruments including the Structured Interview for Prodromal Syndromes and Prodromal Questionnaire.^31^ Interviewers coded each item 0, 1, 2 indicating respectively “not present,” “probably present,” and “definitely present.” All 13 items were summed to create a psychotic experiences scale (range = 0–18, *M* = 1.19, *SD* = 2.58). Just over 30% of participants had at least one psychotic *experience* between ages 12 and 18 (coded 1; *n* = 623, 30.2%), whereas 69.8% reported no psychotic experiences (coded 0; *n* = 1440). These self-reported experiences capture a broader spectrum of more commonly occurring subthreshold psychotic phenomena than psychotic *symptoms* and have not been subject to clinical verification. We additionally examined clinician-verified adolescent psychotic *symptoms* as a secondary outcome, using the same methodology as used at age 12 in this cohort.^6^ Responses to the 7 hallucination/delusion items were verified by a team of clinicians, including child and adolescent psychiatrists, to capture more clinically pertinent psychotic symptoms. Between ages 12 and 18, 2.9% (*n* = 59) of participants were designated as having experienced at least 1 definite psychotic *symptom.* Both measures are described in more detail in the supplementary materials.

To assess suicide attempts, participants were asked whether they had tried to kill themselves or attempted suicide between ages 12 and 18. If they answered positively, further questions about the specific events were asked to obtain details and to establish whether they were accompanied by intent to die. To assess self-harm, participants were asked whether they had ever tried to hurt themselves, to cope with stress or emotional pain. A total of 79 (3.8%) Study members reported a suicide attempt and 280 (13.6%) reported self-harm at age 18. No Study member completed suicide.

Recent evidence suggests that mental health outcomes often correlate with each other.^32^ Therefore, we also tested the effect of childhood psychotic symptoms on the accumulation of poor mental health outcomes in adulthood, by summing all of the dichotomized scores for the different mental health and substance use problems.

### Functional Outcomes in Young Adulthood

#### Psychosocial.

Life satisfaction was assessed by the Satisfaction with Life Scale^33^ and social isolation via the Multidimensional Scale of Perceived Social Support^34^ at age 18. We measured current feelings of loneliness using 4 items from the UCLA Loneliness Scale, version 3.^35^ Participants’ social isolation, low life satisfaction, and loneliness had no predetermined cutoff point, so for these variables we defined poor functioning a priori as being among the 20% highest scoring participants for that outcome.^19^

Sexual behavior was assessed at age 18 with a computer questionnaire based on the 1990 British National Survey of Sexual Attitudes and Lifestyles.^36,37^ The presence of at least 2 of the following items were used to create the risky sexual behavior variable: having had 3 or more sexual partners ever, “never or only sometimes” using contraception, “usually or always” having sex after drugs/alcohol, having contracted a sexually transmitted disease, sexual intercourse before age 16, having had (or caused) a pregnancy. A total of 524 (25.8%) study members reported risky sexual behavior.

Participants were classified as parents if a previous pregnancy had resulted in a live birth or if they were currently pregnant. Girls (2.9%) and boys (1.1%) had experienced or caused at least 1 pregnancy that had resulted in live birth, and 1.7% of girls were pregnant at the age-18 interview. The observed rates match the UK national figures on live births for this age group.^38^

#### Socioeconomic

Low educational attainment was assessed by whether participants did not obtain or scored low (grade D–G) on their General Certificate of Secondary Education (GCSE). GCSEs are a standardized examination taken at the end of compulsory education in the United Kingdom at age 16. Individuals were considered to be not in education, employment or training (NEET) if they reported that they were neither studying, nor working in paid employment, nor pursuing a vocational qualification or apprenticeship training (not due to being on holiday or being a parent). In our cohort, 11.6% (*n* = 239) of participants were NEET, matching UK national figures.^39^

Criminal cautions/convictions were assessed through the UK Police National Computer records searched by the UK Ministry of Justice, and include participants cautioned or convicted in the United Kingdom through age 19 (*n* = 222, 10.8%).

#### Physical Health

Body mass index (BMI) was calculated by dividing weight in kilograms by height in meters’ squared; overweight was defined as a BMI greater than or equal to 25 (*n* = 519, 25.6%). Tobacco dependence (*n* = 183, 8.9%) was assessed using the Fagerstrom Test for Nicotine Dependence.^40^ We measured sleep quality at age 18 years using the Pittsburgh Sleep Quality Index.^41^ It consists of 18 self-report items relating to individuals’ sleep patterns and different forms of sleep impairment in the past month. These questions, scored from 0 to 3, were summed to produce a global score ranging from 0 to 21, with higher scores reflecting worse sleep quality. We adopted the cutoff score of 6 or more as indicative of sleep problems.^42^

A cumulative score was also created by summing the dichotomized functional outcomes for each individual.

### Covariates

To adjust for the potentially confounding effects of individual and family characteristics, comorbid psychopathology, and familial risk, we included as covariates the study participants’ sex, age-5 IQ, family socioeconomic status, participants’ symptoms of depression, anxiety, self-harm/suicidal behavior, ADHD, and conduct disorder at age 12, as well as family psychiatric history and maternal psychotic symptoms. All covariates are described in detail in table 1.

**Table 1.** Descriptive Information for All Covariates Included in the Models

Measure	Age(s) Assessed	Informant	Description	Prevalence *n* (%) or *M* (*SD*)	
				Childhood Psychotic Symptoms Present	Childhood Psychotic Symptoms Absent
Childhood IQ	5	Participant	Vocabulary and Block design subtests on a short form of the Wechsler Preschool and Primary Scale of Intelligence-Revised. Children’s IQs were prorated following procedures described by Sattler^43^	89.1 (14.1)	96.3 (14.4)
Low family socioeconomic status	5	Parents	Participants’ family socioeconomic status was defined using a standardized composite of parents’ income, education, and social class ascertained at childhood phases of the study, which loaded significantly onto one latent factor.^44^ The latent factor was divided in tertiles	53 (42.4)	658 (32.9)
Family history of psychiatric disorder	12	Mother	The Family History Screen was used to assess treatment or hospitalization for a *DSM* psychiatric disorder or substance-use problem or attempted or completed suicide for any of the child’s biological mother, father, grandparents, or aunts and uncles, which was converted to a proportion (0–1.0) of family members with a history of psychiatric disorder^45^	0.5 (0.3)	0.4 (0.3)
Maternal psychosis	12	Mother	Maternal history of psychosis was assessed using the Diagnostic Interview Schedule for *DSM-IV*,^30^ which provides a symptom count for characteristic symptoms of schizophrenia (eg, hallucinations, delusions, anhedonia). This was dichotomized to 0 vs 1 or more	33 (26.6)	317 (15.9)
Depression	12	Participant	Depressive symptoms were assessed using the Children’s Depression Inventory (CDI).^46^ Children who scored 20 or more were deemed to have clinically significant depressive symptoms	12 (9.6)	105 (5.2)
Anxiety	12	Participant	Anxiety symptoms were assessed via private interviews using the 10-item version of the Multidimensional Anxiety Scale for Children (MASC).^47^ An extreme anxiety group was formed with children who scored at or above the 95th percentile	20 (16.0)	107 (5.3)
Attention-deficit hyperactivity disorder (ADHD)	5, 7, 10, 12	Mother Teacher	ADHD was assessed using the *DSM-IV*^28^ and the requirement of symptom onset prior to age 12 was met if parents or teachers reported more than 2 ADHD symptoms at ages 5, 7, 10, or 12 years	5 (4.6)	60 (3.2)
Conduct disorder	12	Mother Teacher	Diagnoses of conduct disorder were based on mothers’ and teachers’ reports of children’s behavior problems using the Achenbach family of instruments and additional *DSM-IV*^28^ items which have previously been described.^48^ Conduct disorder was assumed present if it was diagnosed at ages 5, 7, 10, or 12 years	18 (14.4)	55 (2.7)
Childhood suicidal or self-harm behavior	10, 12	Mother	Report of self-harm or suicide attempt made in past 6 months, at either assessment	11 (8.8)	51 (2.5)

*Note: DSM*, *Diagnostic and Statistical Manual of Mental Disorders*.

### Statistical Analyses

Stata, version 15, was used for all analyses.^49^ We estimated the risk ratios for age-18 mental health and functional outcomes for participants who reported psychotic symptoms at age 12 compared to those who did not, using Poisson regression models. We chose Poisson over logistic regression models to obtain risk ratios for dichotomous outcomes,^50^ which are a more easily interpretable measure of risk particularly when outcomes are relatively common. All of the analyses were corrected for the nonindependence of twin observations using the Huber–White variance estimator.^51^ To test whether the effect of childhood psychotic symptoms on age-18 outcomes was accounted for by the participants’ individual characteristics (sex, childhood IQ), family SES, or other psychopathology at age 12, we included these as potential covariates in our regression models. To take into account the role of measured familial liability to psychopathology, we also included family psychiatric history and maternal psychotic symptoms, in the adjusted models. In addition, we conducted a discordant twin analysis, using fixed-effects models with robust standard errors,^52^ to test the hypothesis that twins with childhood psychotic symptoms would be more likely to have poorer age-18 outcomes than their unaffected co-twins over and above shared family-wide environmental and genetic risk factors.

## Results

### Childhood Psychotic Symptoms and Mental Health Outcomes in Young Adulthood

Participants reporting childhood psychotic symptoms were more likely than those without psychotic symptoms to have poor mental health at age 18 (table 2, model 1). Only 21 of the original 125 individuals (16.8%) with psychotic symptoms at age 12 did not have any of the mental health problems investigated at age 18. Risks were elevated across almost all mental health outcomes, with the exception of PTSD. Individuals with childhood psychotic symptoms were also at higher risk of suicide and/or self-harm, tobacco dependence, and cannabis dependence.

**Table 2.** Childhood Psychotic Symptoms and Risk of Mental Health Problems and Substance Use at Age 18 Years

Young-Adult Outcomes	Model 1^a^		Model 2^b^		Model 3^c^	
	RR (95% CI)	*P* Value	RR (95% CI)	*P* Value	RR (95% CI)	*P* Value
Mental health						
Psychotic experiences	**1.84** (1.51, 2.23)	<**.001**	**1.63** (1.34, 2.00)	<**.001**	**1.44** (1.13, 1.84)	**.003**
Psychotic symptoms	**5.34** (2.92, 9.75)	<**.001**	**5.17** (2.66, 10.04)	<**.001**	**4.14** (1.78, 9.59)	**.001**
Depression	**2.02** (1.55, 2.61)	<**.001**	**2.04** (1.57, 2.64)	<**.001**	**1.76** (1.29, 2.40)	<**.001**
Anxiety	**2.81** (1.84, 4.28)	<**.001**	**2.96** (1.92, 4.56)	<**.001**	**2.41** (1.43, 4.06)	**.001**
Suicide attempts	**5.00** (3.07, 8.16)	<**.001**	**4.84** (3.00, 7.78)	<**.001**	**2.86** (1.42, 5.73)	**.003**
Self-harm	**2.25** (1.60, 3.15)	<**.001**	**2.27** (1.63, 3.15)	<**.001**	**1.61** (1.09, 2.40)	**.017**
PTSD	1.19 (0.54, 2.65)	.661	1.17 (0.54, 2.53)	.694	1.29 (0.53, 3.11)	.577
Conduct disorder	**1.65** (1.16, 2.34)	**.005**	1.33 (0.95, 1.87)	.101	1.19 (0.80, 1.77)	.389
ADHD	**1.99** (1.25, 3.17)	**.004**	**1.62** (1.02, 2.56)	**.040**	1.12 (0.62, 2.02)	.697
Substance use						
Tobacco dependence	**2.53** (1.67, 3.85)	<**.001**	**2.12** (1.39, 3.25)	**.001**	**2.17** (1.38, 3.39)	**.001**
Alcohol dependence	1.26 (0.96, 1.65)	.100	1.27 (0.96, 1.69)	.093	1.12 (0.81, 1.55)	.475
Cannabis dependence	**2.11** (1.22, 3.66)	**.008**	1.66 (0.96, 2.87)	.070	1.60 (0.91, 2.80)	.101
Dependence on other drugs	0.89 (0.13, 5.99)	.904	0.76 (0.11, 5.13)	.779	0.96 (0.20, 4.63)	.964
*Cumulative score for mental health outcomes*	**2.13** (1.76, 2.58)	<**.001**	**1.97** (1.62, 2.38)	<**.001**	**1.65** (1.31, 2.06)	<**.001**

*Note:* The *N* within each model is restricted to participants with non-missing data on all variables included in the multivariate models. The comparison group is those who did not have psychotic symptoms at age 12. Statistically significant results (*P* < .05) are presented in bold text. ADHD, attention-deficit hyperactivity disorder; CI, confidence interval; RR, risk ratio derived using Poisson regression; PTSD, post-traumatic stress disorder. The cumulative score for mental health outcomes was derived by summing all of the dichotomized scores for the different mental health and substance use problems.

^a^Model 1: adjusted for the nonindependence of twin observations.

^b^Model 2: further adjusted for gender, age-5 IQ, and family socioeconomic status.

^c^Model 3: further adjusted for psychopathology at age 12, including depression, anxiety, self-harm/suicidal behavior, ADHD, and conduct disorder.

Adjusting for a range of individual and family characteristics slightly attenuated the associations (table 2, model 2). However, the associations between age-12 psychotic symptoms and age-18 psychotic experiences and symptoms, depression, anxiety, self-injurious behaviors, tobacco dependence, as well as the total number of mental health problems remained statistically significant even after controlling for gender, childhood IQ, family SES, and other childhood psychopathology (table 2, model 3).

### Childhood Psychotic Symptoms and Young-Adult Functional Outcomes

Participants who reported psychotic symptoms at age 12 had poorer psychosocial functioning at age 18 (table 3, model 1), including lower levels of life satisfaction, higher levels of loneliness and social isolation, as well as greater risk of parenthood, risky sexual behaviors, being overweight, sleeping problems, lower educational attainment, and being NEET. However, no significant associations were found with criminal offences by young adulthood. Only 11 of the original 125 individuals (8.8%) with psychotic symptoms at age 12 did not have any of the adverse functional outcomes investigated at age 18.

**Table 3.** Childhood Psychotic Symptoms and Risk of Adverse Functional Outcomes at Age 18 Years

Young-Adult Outcomes	Model 1^a^		Model 2^b^		Model 3^c^	
	RR (95% CI)	*P* Value	RR (95% CI)	*P* Value	RR (95% CI)	*P* Value
Psychosocial						
Low life satisfaction	**1.88** (1.42, 2.49)	<**.001**	**1.78** (1.33, 2.37)	<**.001**	**1.65** (1.19, 2.27)	**.002**
Loneliness	**1.83** (1.48, 2.26)	<**.001**	**1.77** (1.42, 2.19)	<**.001**	**1.44** (1.11, 1.87)	**.005**
Social isolation	**1.65** (1.22, 2.22)	**.001**	**1.56** (1.15, 2.11)	**.004**	**1.41** (1.02, 1.94)	**.035**
Parenthood	**3.46** (1.02, 11.75)	**.046**	**3.57** (1.05, 12.12)	**.041**	**4.97** (1.50, 16.50)	**.009**
Physical health						
Risky sexual behaviors	**1.53** (1.19, 1.97)	**.001**	**1.40** (1.09, 1.80)	**.007**	**1.37** (1.03,1.84)	**.033**
Overweight	1.38 (1.05, 1.81)	**.022**	1.30 (0.99, 1.71)	.063	**1.39** (1.05, 1.85)	**.022**
Sleep problems	**1.42** (1.19, 1.69)	<**.001**	**1.40** (1.18, 1.66)	<**.001**	**1.36** (1.12, 1.66)	**.002**
Socioeconomic						
Low educational attainment	**1.94** (1.50, 2.50)	<**.001**	**1.33** (1.08,1.64)	**.007**	**1.31** (1.01, 1.70)	**.040**
NEET status	**1.87** (1.21, 2.90)	**.005**	1.44 (0.97, 2.14)	.067	1.41 (0.89, 2.22)	.145
Criminal cautions/convictions	1.40 (0.87, 2.25)	.164	0.99 (0.64, 1.53)	.954	1.08 (0.63, 1.84)	.783
Violent offence	1.63 (0.79, 3.37)	.184	1.10 (0.56, 2.16)	.786	1.25 (0.59, 2.66)	.555
Nonviolent offence	1.47 (0.83, 2.60)	.186	1.00 (0.60, 1.68)	.990	1.11 (0.62, 2.00)	.723
*Cumulative score for functional outcomes*	**1.62** (1.37, 1.90)	<**.001**	**1.40** (1.21, 1.63)	<**.001**	**1.36** (1.13, 1.62)	**.001**

*Note:* The *N* within each model is restricted to participants with non-missing data on all variables included in the multivariate models. The comparison group is those who did not have psychotic symptoms at age 12. The cumulative score for functional outcomes was derived by summing all of the dichotomized scores for the different functional outcomes. Statistically significant results (*P* < .05) are presented in bold text. CI, confidence interval; NEET, not in education, employment or training; RR, risk ratio derived using Poisson regression.

^a^Model 1: adjusted for the nonindependence of twin observations.

^b^Model 2: further adjusted for gender, age-5 IQ, and family socioeconomic status.

^c^Model 3: further adjusted for psychopathology at age 12, including depression, anxiety, self-harm/suicidal behavior, attention-deficit hyperactivity disorder, and conduct disorder.

Individual and family characteristics, and other psychopathology at age 12 slightly attenuated the associations between childhood psychotic symptoms and functional outcomes (table 3, models 2–3). Nonetheless, age-12 psychotic symptoms still increased the risk of life dissatisfaction, loneliness and social isolation, parenthood, risky sexual behaviors, being overweight, sleep problems, lower educational attainment, and total number of functional problems at age 18, over and above demographic characteristics and other forms of childhood psychopathology.

### The Role of Genetic and Shared Environmental Factors in the Association Between Childhood Psychotic Symptoms and Outcomes in Young Adulthood

The associations between childhood psychotic symptoms and age-18 mental health and functional outcomes largely remained significant after further adjustment for family history of mental health problems and maternal psychotic symptoms (table 4). However, family psychiatric history seemed to reduce the associations between age-12 psychotic symptoms and social isolation and risky sexual behaviors in young adulthood.

**Table 4.** Childhood Psychotic Symptoms and Risk of Clinical and Functional Problems at Age 18 Years Additionally Adjusted for Family History of Mental Illness

Young-Adult Outcomes	RR (95% CI)	*P* Value
Mental health		
Psychotic experiences	**1.39** (1.09, 1.78)	**.009**
Psychotic symptoms	**3.98** (1.69, 9.36)	**.002**
Depression	**1.69** (1.24, 2.31)	**.001**
Anxiety	**2.29** (1.39, 3.79)	**.001**
Suicide attempt	**2.82** (1.38, 5.73)	**.004**
Self-harm	**1.56** (1.04, 2.36)	**.033**
Substance use		
Tobacco dependence	**2.09** (1.30, 3.35)	**.002**
*Cumulative score for mental health outcomes*	**1.59** (1.27, 2.00)	<**.001**
Psychosocial		
Low life satisfaction	**1.54** (1.11, 2.15)	**.010**
Loneliness	**1.43** (1.11, 1.86)	**.007**
Social isolation	1.36 (0.98, 1.88)	.066
Parenthood	**4.87** (1.38, 17.20)	**.014**
Physical health		
Risky sexual behaviors	1.30 (0.97, 1.75)	.080
Overweight	**1.36** (1.02, 1.83)	**.039**
Sleep problems	**1.34** (1.10, 1.63)	**.004**
*Cumulative score for functional outcomes*	**1.33** (1.11, 1.59)	**.002**

*Note:* This analysis focuses only on those outcomes that were statistically significant at *P* < .05 following adjustment for covariates in tables 2 and 3. Here we adjust for family psychiatric history and maternal psychotic symptoms as well as the nonindependence of twin observations, gender, age-5 IQ, family socioeconomic status, and other psychopathology at age 12. The *N* within each model is restricted to participants with non-missing data on all variables included in the multivariate models. The comparison group is those who did not have psychotic symptoms at age 12. The cumulative score for mental health outcomes was derived by summing all of the dichotomized scores for the different mental health and substance use problems. The cumulative score for functional outcomes was derived by summing all of the dichotomized scores for the different functional outcomes. Statistically significant results (*P* < .05) are presented in bold text. CI, confidence interval; RR, risk ratio derived using Poisson regression.

Most of the associations between childhood psychotic symptoms and poor mental health and functional outcomes at age 18 disappeared when twin pairs within the same family were compared to each other (table 5), suggesting that shared genetic and environmental factors might contribute to these associations. However, associations remained significant for psychotic symptoms, loneliness, and the total number of mental health problems in young adulthood, indicating that the association of childhood psychotic symptoms with these outcomes was largely independent of family-wide risk factors.

**Table 5.** The Effect of Unmeasured Familial Risk Factors in the Associations Between Childhood Psychotic Symptoms and Clinical and Functional Outcomes at Age 18 Years Within Twins in the Same Family

Age-18 Outcomes	Fixed-Effects Bivariate Model	
	RR (95% CI)	*P* Value
Psychotic experiences	1.17 (0.86,1.58)	.318
Psychotic symptoms	**9.00** (1.14, 71.04)	**.037**
Depression	1.36 (0.87, 2.11)	.172
Anxiety	1.78 (0.87, 3.62)	.113
Suicide attempt	1.67 (0.64, 4.29)	.290
Self-harm	1.06 (0.63, 1.77)	.827
Tobacco dependence	1.00 (0.57, 1.75)	1.000
*Cumulative score for mental health outcomes*	**1.36** (1.02, 1.81)	**.038**
Low life satisfaction	1.53 (0.98, 2.37)	.061
Loneliness	**1.82** (1.25, 2.64)	**.002**
Parenthood	0.50 (0.04, 5.51)	.571
Risky sexual behaviors	1.30 (0.90, 1.89)	.163
Overweight	1.32 (0.91, 1.91)	.146
Sleep problems	1.09 (0.84, 1.43)	.505
*Cumulative score for functional outcomes*	1.15 (0.95, 1.39)	.153

*Note:* The *N* within each model is restricted to participants with non-missing data on all variables included in the multivariate models. The comparison group is those who did not have psychotic symptoms at age 12. The cumulative score for mental health outcomes was derived by summing all of the dichotomized scores for the different mental health and substance use problems. The cumulative score for functional outcomes was derived by summing all of the dichotomized scores for the different functional outcomes. Statistically significant results (*P* < .05) are presented in bold text. CI, confidence interval; RR, risk ratio.

## Discussion

Psychotic symptoms in childhood are a well-known risk factor for clinically relevant psychosis later in life.^53^ Using a nationally representative prospectively assessed longitudinal birth cohort, we showed strong evidence of continuity between early psychotic symptoms in childhood and persistence of such symptoms to young adulthood over and above shared genetic and environmental influences. Our results are in line with previous findings showing evidence of a longitudinal relationship between early psychotic symptoms and clinically relevant psychosis in adulthood^1^ and support the hypothesis of a continuum of psychotic phenomena.^54^

However, children with psychotic symptoms were more likely to experience a higher number of mental health problems in young adulthood compared to children without such symptoms including a broad spectrum of nonpsychotic psychopathology. This is in keeping with previous studies,^7,11,55^ and supports the hypothesis that early psychotic phenomena are part of a latent continuum of common mental distress.^56^ In addition, childhood psychotic symptoms were also associated with a wide array of poor functional outcomes, leading to high personal and societal burden. It is important to note though that our results did not confirm an association between childhood psychotic symptoms and later criminal offending/antisocial behavior. These findings support the presence of different developmental pathways underlying delinquent behaviors in young adulthood,^57^ that might be characterized by the interplay of polygenic risk, conduct disorder, childhood victimization, lower cognitive abilities, and poor self-control.^58^ Overall, we extended existing findings by documenting that psychotic symptoms in childhood predicted a broad range of clinically relevant and functional outcomes in young adulthood over and above other forms of childhood psychopathology, and individual and family characteristics.

Our results are similar to findings from the Dunedin cohort, which also found that only a small minority of the children presenting with age-11 psychotic symptoms were free from *DSM* disorders by age 38.^8^ Therefore, children with psychotic symptoms appear to struggle to make an optimal transition to adulthood, which may have a detrimental impact on their well-being, social relationships, and productivity throughout their adult years.

As these early psychotic phenomena appear to herald a multitude of mental health and functional problems at the crucial stage of transitioning to adulthood, this further highlights the importance of detecting psychotic symptoms early and intervening swiftly to steer the child away from such adverse developmental trajectories.^59^ However, our twin analyses indicated that most of the associations between childhood psychotic symptoms and poor outcomes in young adulthood were explained by familial factors suggesting that early psychotic phenomena could not be considered to be causing later problems (aside potentially from psychotic symptoms, loneliness, and the total number of mental health problems in young adulthood which remained associated when familial factors were taken into account).

This does not detract from the potential usefulness of screening children for psychotic symptoms to identify those at risk, but does indicate that interventions to prevent later adverse outcomes among such children would need to be targeted at genetic and environmental factors shared within families rather than at the psychotic symptoms themselves. Further research is required to elucidate these familial risk factors to inform preventive interventions. Given that we found psychotic symptoms predicted later adverse outcomes over and above other forms of psychopathology in childhood, implementing routine screening for psychotic symptoms in child and adolescent services may assist clinicians to identify which children presenting with common emotional and behavioral problems are most at risk of poor psychopathological and functional outcomes in young adulthood and thus require additional interventions to minimize these adverse outcomes, such as educational support services. As a large proportion of children with mental health problems are not seen by psychiatric services (or at least not quickly) it will be important to consider how best to detect psychotic symptoms among children in the general population and provide interventions to promote positive outcomes in order to have a substantial impact. This is a worthwhile enterprise in order to potentially prevent the onset of enduring mental health problems and lifelong disability in these vulnerable children but will require careful investigation.

### Limitations

Our findings should be interpreted in light of some limitations. First, the prevalence of childhood psychotic symptoms was low and this may have reduced the possibility to detect associations with some of the clinical and functional outcomes. We purposely chose a conservative approach to defining the presence of psychotic symptoms in childhood to ensure we captured only clinically relevant phenomena in order to optimally target future preventive intervention strategies without potentially stigmatizing a large number of children. Furthermore, we focused on 7 childhood positive psychotic symptoms related mainly to delusions and hallucinations. Therefore, our assessment did not capture other relevant psychotic phenomena, such as thought disorder, negative symptoms, and cognitive disorganization. However, our questions have been validated and used in other prospective cohort studies to screen for psychotic symptoms.^21,27,60^

Second, although childhood psychotic symptoms predicted poor adult outcomes our findings indicate that these symptoms largely reflect familial genetic and environmental risks for poor outcomes rather than being the cause of these outcomes. This finding does not undermine the prognostic significance of childhood psychotic symptoms but indicates that merely reducing the occurrence of these symptoms will not improve mental health and functional outcomes in young adulthood. It might also be that other environmental factors not shared between twins, such as school experiences and traumatic events in childhood^61^ or other life events, explain many of the observed associations. This requires investigation in future studies.

Third, we focused on outcomes only at age 18 and it is possible that poorer outcomes might be detected later in life. Nevertheless, young adulthood is a crucial phase of life when individuals are transitioning to independence from their families of origin to forge romantic partnerships and develop their own families, as well as typically undertaking qualifications or apprenticeships that will shape their future earning power and capacity to contribute economically to society.

Fourth, we were not able to conduct more sophisticated analyses to disentangle the effect of latent genetic and environmental factors on the associations between childhood psychotic symptoms and later adverse outcomes. The modest number of MZ (*n* = 33) and DZ (*n* = 36) twin pairs with age-12 psychotic symptoms in this sample prevented us from conducting structural equation modeling, which typically requires at least 200 twin pairs.^62^ However, we were able to use a discordant twin analysis to account for shared environmental and genetic effects on these associations, which has the advantage of being easier to communicate to a nonspecialist audience.

Finally, our results may not be generalizable to singletons as we studied a cohort of twins. Twins have a lower birth weight and are born on average approximately 3 weeks preterm compared to singletons.^63^ Obstetric complications also represent an important risk factor for schizophrenia^64^ and, therefore, twins might be exposed to increased developmental risk factors for psychosis. However, the prevalence of psychotic symptoms in our cohort is comparable with the ranges estimated in samples of singletons.^2,27^ It is also important to note that the E-Risk study members have been followed up to age 18 with a 93% retention rate and, therefore, our results are unlikely to be explained by attrition. Nonetheless, our findings may have limited generalizability to other countries, particularly low-income countries, where prevalence rates of subthreshold psychotic phenomena have been shown to differ.^65^

## Conclusions

Our findings indicate that childhood psychotic symptoms are an early indicator of a range of mental health problems, self-injurious and physically harmful behaviors, obesity, and poor social functioning at the crucial transition to adulthood, independent of the potentially confounding effects of sex, socioeconomic deprivation, low IQ, and comorbid psychopathology. These early symptoms may, therefore, act as a useful way of identifying children who are at risk for an array of poor outcomes in young adulthood and who may benefit from preventive interventions. However, many of the associations between childhood psychotic symptoms and poor outcomes were explained by familial risk factors indicating that such interventions would need to be targeted at these factors rather than the psychotic symptoms themselves.

## Funding

The E-Risk Study is funded by the UK Medical Research Council (G1002190). Additional support was provided by the National Institute of Child Health and Human Development (HD077482); the Jacobs Foundation; a National Institute of Health Research (NIHR) Maudsley BRC Preparatory Clinical Research Training Fellowship to A.T.; and an MQ Fellows Award (MQ14F40) and a British Academy Mid-Career Fellowship (MD\170005 to H.L.F.). L.A. is the Mental Health Leadership Fellow for the UK Economic and Social Research Council. C.P. was supported by the NIHR Biomedical Research Centre at the South London and Maudsley NHS Foundation Trust and King’s College London. The views expressed are those of the authors and not necessarily those of the National Health Service, the National Institute for Health Research, or the Department of Health and Social Care.

## Supplementary Material

sbz069_suppl_Supplementary-Material
