# Supplementary material for: Mental Health and Functional Outcomes in Young Adulthood of Children With Psychotic Symptoms: A Longitudinal Cohort Study
Source: Schizophr Bull. 2019 Jul 30;46(2):261–71. doi: 10.1093/schbul/sbz069 (PMC7442396; doi:10.1093/schbul/sbz069)
Supplement: sbz069_suppl_Supplementary-Material [file sbz069_suppl_supplementary-material.docx]

**Supplementary Materials**

*Trotta et al “Mental health and functional outcomes in young adulthood of children with psychotic symptoms: a longitudinal cohort study”*

**Supplementary Methods**

***Measure of childhood psychotic symptoms***

E-Risk families were visited by mental health trainees or professionals when children were aged 12.^1^ Each child was privately interviewed about 7 psychotic symptoms pertaining to delusions and hallucinations, with items including “have other people ever read your thoughts?,” “have you ever thought you were being followed or spied on?,” and “have you ever heard voices that other people cannot hear?.” This interview has been described in detail previously.^1^ The item choice was guided by the Dunedin Study’s age-11 interview protocol^2^ and an instrument prepared for the Avon Longitudinal Study of Parents and Children.^3^ Interviewers coded each experience 0, 1, 2 indicating respectively “not a symptom,” “probable symptom,” and “definite symptom.” A conservative approach was taken in designating a child’s report as a symptom. First, the interviewer probed using standard prompts designed to discriminate between experiences that were plausible (e.g., “I was followed by a man after school”) and potential symptoms (e.g., “I was followed by an angel who guards my spirit”), and wrote down the child’s narrative description of the experience. Second, items and interviewer notes were assessed by a psychiatrist expert in schizophrenia, a psychologist expert in interviewing children, and a child and adolescent psychiatrist to verify the validity of the symptoms. Third, because children were twins, experiences limited to the twin relationship (e.g., “My twin and I often know what each other are thinking”) were coded as “not a symptom”. Children were only designated as experiencing psychotic symptoms if they reported at least one definite symptom. At age 12, 5.9% (*N* = 125) of children reported experiencing psychotic symptoms. This is similar to the prevalence of psychotic symptoms in other community samples of children and adolescents.^4-8^ Furthermore, we have previously shown that childhood psychotic symptoms in this cohort have good construct validity, sharing many of the genetic, social, neurodevelopmental, and behavioral risk factors and correlates as adult schizophrenia.^1^

***Measure of psychotic symptoms in young adulthood***

The same items and clinical verification procedure as above was used when participants were interviewed at age 18, this time enquiring about psychotic symptoms they may have experienced since age 12. At age 18, 2.9% (N=59) of participants reported experiencing psychotic symptoms since age 12 that were clinically verified (referred to as young adult psychotic symptoms).

***Measure of psychotic experiences in young adulthood***

To obtain a broader measure of young adult psychotic experiences during the age 18 interviews, participants were asked six items about unusual feelings and thoughts in addition to the seven hallucination/delusion items. These items drew on item pools since formalised in prodromal psychosis screening instruments including the Prevention through Risk Identification, Management and Education (PRIME)-screen^9^ and the Structured Interview for Psychosis-Risk Syndromes (SIPS).^10^ These additional items included: I have become more sensitive to lights or sounds; I feel as though I can’t trust anyone; I worry that my food may be poisoned; People or places I know seem different; I believe I have special abilities or powers beyond my natural talents; My thinking is unusual or frightening. Interviewers coded each of the 13 items (7 original hallucination/delusion items plus 6 additional unusual experiences items) 0, 1, 2, indicating respectively “not present”, “probably present” and “definitely present”. Responses to each of the 13 items (none, probable, definite) were summed to create a psychotic experiences scale (potential range=0–26, actual range=0–18, M=1.19, SD=2.58). The psychotic experiences measure did not involve clinical verification, meaning that this is a self-report measure capturing a broader range of mild, moderate and potentially clinically pertinent hallucinations, delusions, and other unusual feelings and thoughts. Since there were low numbers of adolescents with high psychotic experiences scores (e.g., only 1.0% [N=21] of participants had a psychotic experiences score of 13 or more), scores were placed into an ordinal scale to tackle the skewed distribution while retaining more information than a binary score. Just over 30% of participants had at least one psychotic experience between ages 12 and 18: 69.8% reported no psychotic experiences (coded 0; N=1,440), 15.5% reported 1 or 2 psychotic experiences (coded 1; N=319), 8.1% reported 3–5 psychotic experiences (coded 2: N=166), and 6.7% reported 6 or more psychotic experiences (coded 3: N=138). This 30.2% prevalence is similar to the prevalence of self-reported psychotic experiences in other community samples of teenagers and young adults.^11-13^

**References**

1. Polanczyk G, Moffitt T, Arseneault L, et al. Etiological and clinical features of childhood psychotic symptoms. *Arch Gen Psychiatry.* 2010;67(4):328-338.

2. Poulton R, Caspi A, Moffitt TE, et al. Children’s self-reported psychotic symptoms and adult schizophreniform disorder: a 15-year longitudinal study. *Arch Gen Psychiatry.* 2000;57:1053-1058.

3. Schreier A, Wolke D, Thomas K, et al. Prospective study of peer victimization in childhood and psychotic symptoms in a non-clinical population at age 12 years. *Arch Gen Psychiatry.* 2009;66:527-536.

4. Kelleher I, Connor D, Clarke MC, Devlin N, Harley M, Cannon M. Prevalence of psychotic symptoms in childhood and adolescence: a systematic review and meta-analysis of population-based studies. *Psychol Med.* 2012;42:1857-1863.

5. Horwood J, Salvi G, Thomas K, et al. IQ and non-clinical psychotic symptoms in 12-year-olds: results from the ALSPAC birth cohort. *Br J Psychiatry.* 2008;193(3):185-191.

6. Yoshizumi T, Murase S, Honjo S, Kaneko H, Murakami T. Hallucinatory experiences in a community sample of Japanese children. *J Am Acad Child Adolesc Psychiatry.* 2004;43(8):1030-1036.

7. Scott J, Chant D, Andrews G, McGrath J. Psychotic-like experiences in the general community: the correlates of CIDI psychosis screen items in an Australian sample. *Psychol Med.* 2006;36(2):231-238.

8. Dhossche D, Ferdinand R, Van der Ende J, Hofstra MB, Verhulst F. Diagnostic outcome of self-reported hallucinations in a community sample of adolescents. *Psychol Med.* 2002;32:619-627.

9. Miller TJ, Cicchetti D, Markovich PJ, McGlashan TH, Woods SW. The SIPS-Screen: a brief self-report screen to detect the schizophrenia prodrome. *Schizophr Res*. 2004;70 (suppl1):78.

10. Miller TJ, McGlashan TH, Rosen JL, et al. Prodromal assessment with the structured interview for prodromal syndromes and the scale of prodromal symptoms: predictive validity, interrater reliability, and training to reliability. *Schizophr Bull*. 2003;29(4):703.

11. Spauwen J, Krabbendam L, Lieb R, Wittchen HU, van Os J. Does urbanicity shift the population expression of psychosis? *J Psychiatr Res*. 2004;38(6):613-618.

12. Yoshizumi T, Murase S, Honjo S, Kaneko H, Murakami T. Hallucinatory experiences in a community sample of Japanese children. *J Am Acad Child Adolesc Psychiatry*. 2004;43(8):1030-1036.

13. Yung AR, Nelson B, Baker K, et al. Psychotic-like experiences in a community sample of adolescents:

Implications for the continuum model of psychosis and prediction of schizophrenia. *Aust N Z J Psychiatry*. 2009;43(2):118-128.
